# Comparative proteomic profiling reveals a role for Cisd2 in skeletal muscle aging

**DOI:** 10.1111/acel.12705

**Published:** 2017-11-23

**Authors:** Yi‐Long Huang, Zhao‐Qing Shen, Chia‐Yu Wu, Yuan‐Chi Teng, Chen‐Chung Liao, Cheng‐Heng Kao, Liang‐Kung Chen, Chao‐Hsiung Lin, Ting‐Fen Tsai

**Affiliations:** ^1^ Department of Life Sciences and Institute of Genome Sciences National Yang‐Ming University Taipei Taiwan; ^2^ Program in Molecular Medicine School of Life Sciences National Yang‐Ming University and Academia Sinica Taipei Taiwan; ^3^ Proteomics Research Center National Yang Ming University Taipei Taiwan; ^4^ Center of General Education Chang Gung University Taoyuan Taiwan; ^5^ Center for Geriatrics and Gerontology Taipei Veterans General Hospital Taipei Taiwan; ^6^ Aging and Health Research Center National Yang‐Ming University Taipei Taiwan; ^7^ Genome Research Center National Yang‐Ming University Taipei Taiwan; ^8^ Institute of Molecular and Genomic Medicine National Health Research Institutes Zhunan Taiwan

**Keywords:** calcium homeostasis, Cisd2, ER stress, gastrocnemius muscle, proteomics, Serca

## Abstract

Skeletal muscle has emerged as one of the most important tissues involved in regulating systemic metabolism. The gastrocnemius is a powerful skeletal muscle composed of predominantly glycolytic fast‐twitch fibers that are preferentially lost among old age. This decrease in gastrocnemius muscle mass is remarkable during aging; however, the underlying molecular mechanism is not fully understood. Strikingly, there is a ~70% decrease in Cisd2 protein, a key regulator of lifespan in mice and the disease gene for Wolfram syndrome 2 in humans, within the gastrocnemius after middle age among mice. A proteomics approach was used to investigate the gastrocnemius of naturally aged mice, and this was compared to the autonomous effect of Cisd2 on gastrocnemius aging using muscle‐specific Cisd2 knockout (mKO) mice as a premature aging model. Intriguingly, dysregulation of calcium signaling and activation of UPR/ER stress stand out as the top two pathways. Additionally, the activity of Serca1 was significantly impaired and this impairment is mainly attributable to irreversibly oxidative modifications of Serca. Our results reveal that the overall characteristics of the gastrocnemius are very similar when naturally aged mice and the Cisd2 mKO mice are compared in terms of pathological alterations, ultrastructural abnormalities, and proteomics profiling. This suggests that Cisd2 mKO mouse is a unique model for understanding the aging mechanism of skeletal muscle. Furthermore, this work substantiates the hypothesis that Cisd2 is crucial to the gastrocnemius muscle and suggests that Cisd2 is a potential therapeutic target for muscle aging.

## 1 INTRODUCTION

Sarcopenia, characterized by a progressive loss of skeletal muscle mass and function, is one of the most prominent features during aging. Skeletal muscle fibers can be classified into different types based on their physiological and metabolic characteristics. Type I muscle fibers are slow‐twitch fibers; these fibers are associated with high levels of oxidative enzyme activity, low levels of glycolytic enzyme activity, large number of mitochondria, and slow‐twitch contraction. Type II muscle fibers are fast‐twitch fibers; these fibers are associated with high levels of glycolytic enzyme activity, fewer mitochondria, and fast contraction times. Importantly, these different subtypes of muscle fibers exhibit different sensitivities to aging. Previous studies have revealed that there is preferential loss and atrophy of glycolytic fast‐twitch fibers compared to slow‐twitch muscle fibers on aging (Lexell, 1995; Thompson, 1994). Interestingly, two studies have shown that fast‐twitch muscle fiber‐specific restoration is able to help regulate glucose metabolism and adipose tissue homeostasis in mice (Akasaki et al., 2014; Tsai et al., 2015), which implies that the fast‐twitch muscles have an important role during aging.

The gastrocnemius is a powerful superficial bipennate muscle located with the soleus in the posterior (back) compartment of the lower leg in humans and in the hind leg in mice. Decreases in the muscle mass of the gastrocnemius, composed of predominantly fast‐twitch fibers, are quite remarkable during aging. In contrast, the soleus muscle, composed of predominantly slow‐twitch fibers, shows less atrophy and is much less affected during aging among humans and rodents (Braga et al., 2008; Kovacheva, Hikim, Shen, Sinha & Sinha‐Hikim, 2010; Martin et al., 2007; Sinha‐Hikim et al., 2013). This is consistent with observations indicating that fiber size decline prominently affects fast‐twitch fibers, and that slow‐twitch fibers are less affected. However, the molecular mechanisms underlying the degeneration and dysfunction of the gastrocnemius muscle during aging are not fully understood.

The CDGSH iron‐sulfur domain‐containing protein 2 (CISD2) plays a crucial role in lifespan control and human disease. Recessive mutations in human CISD2 cause type 2 Wolfram syndrome (WFS2; MIM 604928), a rare neurodegenerative and metabolic disorder associated with a shortened lifespan. In the Cisd2 knockout mice, Cisd2 deficiency shortens lifespan and drives premature aging; additionally, neuronal lesions and muscle abnormalities are the two earliest manifestations of this premature aging phenotype, and these precede the gross premature aging phenotype (Chen et al., 2009). The Cisd2 protein has been detected in various different subcellular localities, being enriched in the mitochondrial outer membrane fraction, in the ER, and in the mitochondria‐associated ER membranes (MAMs) of various cell types (Wang, Kao, Chen, Wei & Tsai, 2014). ER and mitochondria are the two major intracellular calcium stores that respond to signals for calcium mobilization, while MAMs serve as hotspots for calcium transfer between the ER and mitochondria. Previous studies had revealed that Cisd2 plays an essential role in mitochondrial integrity and in the regulation of intracellular calcium homeostasis (Chang et al., 2012; Chen, Wu, Kirby, Kao & Tsai, 2010; Lu et al., 2014; Wang, Chen, et al., 2014; Wiley et al., 2013).

So far, few proteomics studies have been reported that involving premature muscle degeneration. Accordingly, it is anticipated that a detailed analysis and comparison of the differentially and commonly expressed proteins between naturally aged and Cisd2 mKO muscle tissues may lead to the discovery of critical aging regulators rather than disease‐specific and age‐related protein effects. In this study, we took a proteomics approach followed by biochemical validation; the aim was to investigate age‐related protein changes within the gastrocnemius muscle. Our intent was to identify the top altered pathways and explore their association with pathological alterations. We generated muscle‐specific Cisd2 KO (mKO) mice to investigate the autonomous effect of Cisd2 on the gastrocnemius muscle. Furthermore, proteomic profiling of naturally aged wild‐type (WT) mice was also carried out and these results compared with those from young WT and Cisd2 mKO mice. The target was to address the biological relevance of Cisd2 deficiency to the aging of the gastrocnemius muscle.

## 2 RESULTS

### 2.1 Age‐dependent decrease in Cisd2 levels and degeneration of gastrocnemius muscle in the naturally aged mice and Cisd2 mKO mice

**Figure 1:**
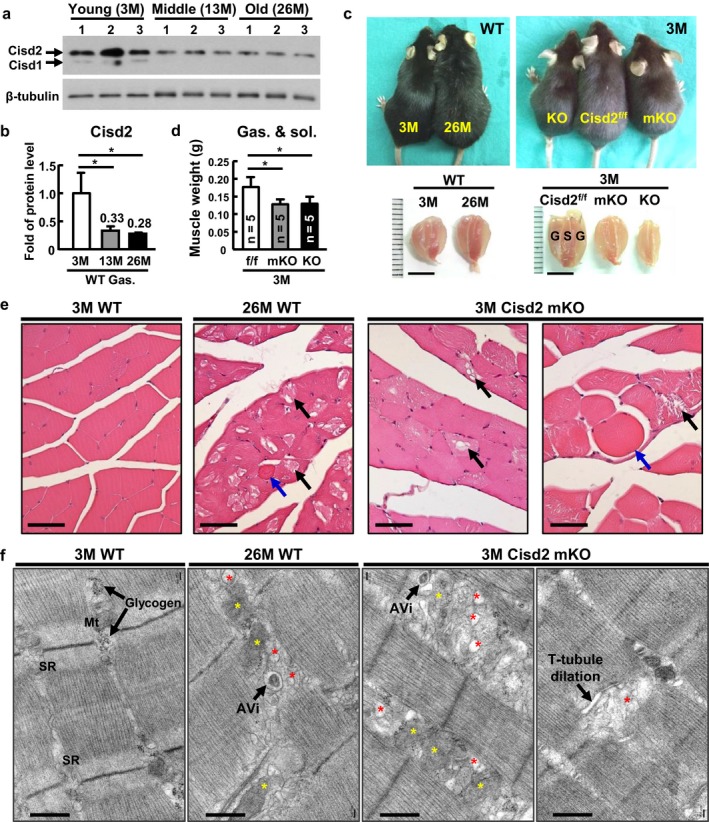
Age‐dependent decrease in Cisd2 within the gastrocnemius of WT mice and the pathological defects found in the gastrocnemius of naturally aged mice and Cisd2 mKO mice. (a,b) Decrease in Cisd2 protein level in the gastrocnemius muscle at middle age, 13‐month‐old (13M) and old age (26M) compared with young (3M) WT mice. (c) Gross view of the mice and of the gastrocnemius muscles for naturally aged mice (3M vs. 26M WT mice), and Cisd2 genetically modified mice (systemic Cisd2 KO, Cisd2^f/f^ and mKO mice). S, soleus; G, gastrocnemius. (d) Quantification of muscle weight of the gastrocnemius. (e) H&E staining of transverse sections of the gastrocnemius muscle of 3M WT, 26M WT, and 3M Cisd2 mKO mice. The black arrows indicate degenerative loss of muscle fibers. The blue arrows indicate the rounded fibers. Scale bars, 50 μm. (f) Ultrastructural alterations in the gastrocnemius muscles of 3M WT, 26M WT, and 3M Cisd2 mKO mice as revealed by TEM. The red stars indicate dilated/degenerate SR. The yellow stars indicate degenerate mitochondria. Early or initial autophagic vacuoles (AVi), namely autophagosomes, which are double‐membrane structures containing undigested cytoplasmic organelles, were detected in the 26M WT and 3M Cisd2 mKO mice. Mt, mitochondria. Scale bars, 500 nm

Previously, we have shown that there is an age‐dependent decrease in Cisd2 expression levels within the brain and the femoris muscle using C57BL/6 WT mice (Chen et al., 2010; Wu et al., 2012). The mean lifespan of C57BL/6 mice is 25.5 ± 3.9 months (*n* = 50) in our mouse facility. In the femoris muscle, there was an average 38% and 57% decrease in the Cisd2 protein level during middle age (12‐month‐old [12M]) and during old age (24M), respectively, compared with young (3M) mice (Wu et al., 2012). Strikingly, in the gastrocnemius, there was a much higher decline, namely a ~70% decrease in Cisd2 protein level during middle/old age (Figure 1a,b). This suggests that Cisd2 expression in the gastrocnemius is more sensitive to aging and such reduced protein levels are likely to impact on the age‐related alterations that affect the gastrocnemius.

Cisd2 mKO (MCK‐Cre; Cisd2^f/f^) mice were used to study the biological effects of Cisd2 deficiency on the skeletal muscle and exclude potential nonautonomous effects from nonmuscle tissues (Fig. S1). The weights of the gastrocnemius and soleus muscles, which are tightly associated, seemed to decrease to a similar extent during systemic and muscle‐specific Cisd2 knockout (Figure 1c,d). Pathologically, naturally aged (26M WT) mice displayed overt degenerative loss and occasional rounded and shrunken fibers in the gastrocnemius (Figure 1e left panel). These pathological alterations were also detectable in Cisd2 mKO mice at 3M (Figure 1e right panel). Ultrastructurally, in the naturally aged mice, we found dilated SR and degenerate intermyofibrillar mitochondria along with autophagic vacuoles. In the Cisd2 mKO mice, there were more severe alterations in the mitochondria and SR; additionally, T‐tubule dilation also could be detected in the prematurely aged gastrocnemius (Figure 1f).

### 2.2 Proteomic profiling of the gastrocnemius muscles in the naturally aged and Cisd2 mKO mice

**Figure 2:**
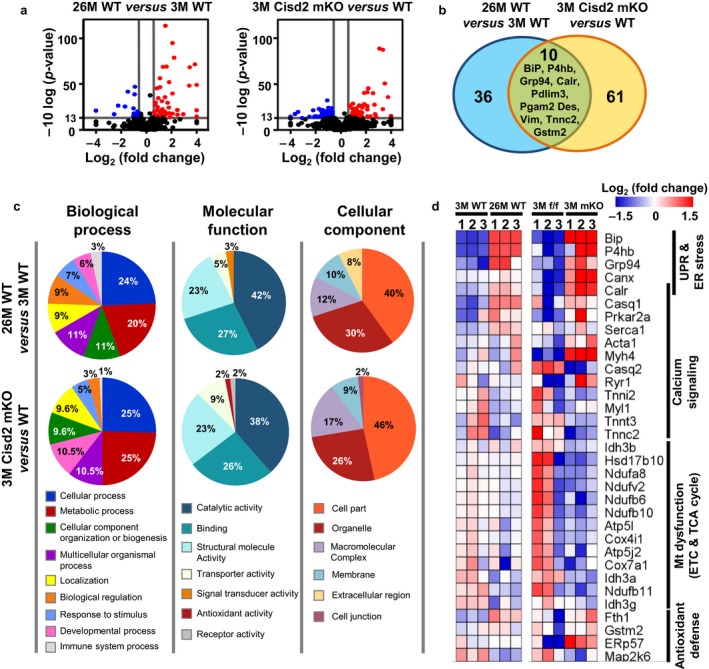
Identification and functional classification of the differentially expressed proteins (DEPs) present in the gastrocnemius of naturally aged mice and Cisd2 mKO mice. (a) Volcano plots showing the selection of DEPs based on fold change and *p‐*value for the comparisons of old (26M) vs. young (3M), and 3M Cisd2 mKO vs. WT (Cisd2^f/f^). The horizontal lines denote the cutoff threshold for significance (*p‐*value < .05, −10 log *p *>* *13). The vertical lines denote the threshold for fold change (1.5‐fold change, namely log_2_ (fold change) = 0.585). Spots that pass the criteria are shown in either red (increased) or blue (decreased). (b) A Venn diagram illustrating the common and unique DEPs in the gastrocnemius between old *vs*. young and Cisd2 mKO vs. WT (Cisd2^f/f^) mice. The numbers represent the proportion of significantly changed proteins (fold change > 1.5, *p *<* *.05) in each pair wise comparison and in their respective overlaps. (c) A pie chart of the distribution of DEPs according to their biological processes, molecular functions, and subcellular localization based on Gene Ontology annotation. The categorizations were carried via the PANTHER online tool system. (d) Pathway‐level heat map illustrating the significant canonical pathways that are related to Cisd2 function, as determined by IPA. Complete dataset can be found in Table S2

To obtain a better understanding for the mechanism underlying the age‐related alterations of the gastrocnemius, a label‐free proteomics approach using LC‐MS/MS was applied to investigate the protein changes in the following two mouse groups: (i) 3M Cisd2 mKO vs. 3M Cisd2^f/f^ control and (ii) 26M old vs. 3M young WT (Fig. S2a). The PEAKS, a software program developed recently with improved sensitivity and accuracy, was used to quantify the identified proteins by LC‐MS/MS. With 1% false discovery rate (FDR), a total of 865 and 1,021 proteins were identified in the first group (Cisd2 mKO vs. Cisd2^f/f^) and second group (old WT vs. young WT), respectively. As the protein quantification algorithm relies on the extracted ion chromatogram (XIC) of unique peptides, we only selected proteins with ≥2 unique peptides for further label‐free quantification analysis. The differentially expressed proteins (DEPs) were defined those with a significance >13 (*p *<* *.05) and a fold change >1.5 compared to their control group. A total of 46 and 71 proteins were, respectively, recognized as DEPs in the naturally aged mice and Cisd2 mKO mice (Figure 2a; Table S1). Furthermore, 10 proteins were observed in both DEP datasets (BiP, Grp94, Calr, P4hb, Pdlim3, Pgam2, Des, Vim, Gstm2, and Tnnc2) (Figure 2b; Figs S3, S4). Notably, four of 10 of these common DEPs (Calr, Grp94, BiP, and P4hb) were all up‐regulated and involved in protein maturation and ER stress. This implies that these proteins probably have a critical role in proteostasis during the aging process. Moreover, Gene Ontology classification revealed that most DEPs in both datasets are involved in similar annotations, namely biological process, molecular function or cellular component (Figure 2c), indicating the presence of commonly altered functional pathways within the gastrocnemius in the naturally aged mice and Cisd2 mKO mice.

To further identify and compare the top altered pathways between the two aging models, these DEPs were investigated by Ingenuity Pathway Analysis (IPA). Statistically significant canonical pathways enriched in the two DEP datasets at *p *<* *.01 were identified (Table S2). Among these, the calcium signaling and unfolded protein response (UPR)/ER stress pathways, which are highly associated with the function of Cisd2, stand out among the topmost pathways altered in both the naturally aged and Cisd2 mKO mice. By way of contrast, only the Cisd2 mKO mice showed significant mitochondrial dysfunction. Furthermore, previous studies have revealed that Cisd2‐deficient mouse embryonic fibroblasts (MEFs) exhibit increased levels of reactive oxygen species (ROS) and reactive nitrogen species (RNS) (Wiley et al., 2013), and that Cisd2 overexpression is able to protect cells from H_2_O_2_‐induced oxidative stress and cell death using a breast cancer cell model (Darash‐Yahana et al., 2016). In this context, four DEPs that are related to the ROS response and redox regulation were pinpointed; these are involved in the “NRF2‐mediated oxidative stress response” and “glutathione redox reactions” pathways. The expression profiles of the above‐mentioned DEPs, provided in Figure 2d as a heat map, along with the involved pathways or antioxidant function, form an interesting group of pathways associated with muscle aging.

### 2.3 Activation of UPR/ER stress stands out as the top pathway above all the other pathways enriched in the DEP datasets

**Figure 3:**
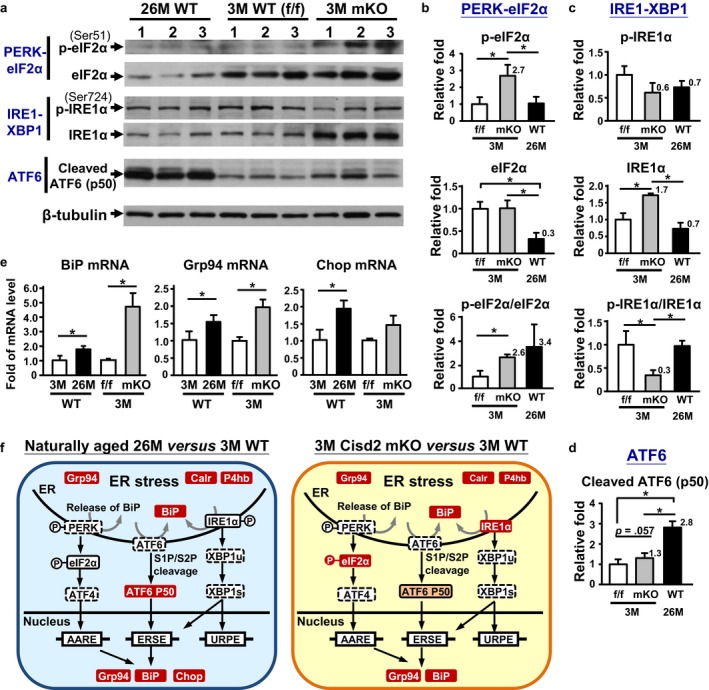
Activation of UPR and ER stress in the naturally and prematurely aged gastrocnemius muscle. (a) Western blot analyses of the three UPR pathway markers, namely PERK‐eIF2α, IRE1‐XBP1, and ATF6. β‐Tubulin in the ATF6 blot is shown as the representative internal control. See Fig. S5b–d for three complete blots. (b) Quantification of the p‐eIF2α and total eIF2α and the ratio of p‐eIF2α/total eIF2α. *p <* *.05. (c) Quantification of the p‐IRE1α and total IRE1α and the ratio of p‐IRE1α/total IRE1α. (d) Quantification of the cleaved ATF6 (p50). (e) Up‐regulation of the mRNA expression levels of the three UPR downstream genes (BiP, Grp94, and Chop) determined by real‐time RT‐qPCR. The expression levels were normalized to hypoxanthine guanine phosphoribosyl transferase (*Hprt*). *n* = 3 for each group. (f) Schematic illustration of the ER stress and UPR pathways in the gastrocnemius muscles of naturally aged WT and Cisd2 mKO mice. Red color indicates up‐regulated DEPs with a statistical significance, and orange color indicates proteins showing an increasing trend (*p *<* *.1)

To further ascertain whether ER stress and the UPR signaling pathway are indeed activated in the two aging models, we used Western blotting to validate three UPR transducers, namely eIF2α, ATF6α, and IRE1α. Our results revealed that the ATF6 arm of the UPR was selectively activated in the age‐related ER stress of old (26M) WT mice, whereas the PERK/eIF2α arm of the UPR was strongly activated in the 3M Cisd2 mKO (Figure 3a–d). Interestingly, while the protein level of phosphorylated‐IRE1α remained unchanged, the total protein level of IRE1α was significantly increased in the Cisd2 mKO gastrocnemius (Figure 3a,c). Furthermore, the mRNA levels of UPR downstream target genes, BiP, Grp94, and Chop, were also demonstrated to be activated (Figure 3e). These results are consistent with the pathological findings of dilated/degenerated SR (Figure 1f; Fig. S5a), which further supports our earlier proteomic and biochemical evidence. Taken together, both naturally and prematurely aged gastrocnemius muscles exhibited ER stress as revealed by activation of one of the three UPR upstream sensors and induction of downstream target gene expression. A graphical summary comparing the ER stress and UPR pathways between the two mouse aging models is presented in Figure 3f.

### 2.4 Dysregulation of calcium homeostasis is involved in the natural and premature aging of the gastrocnemius muscle, but this is associated with different DEPs

**Figure 4:**
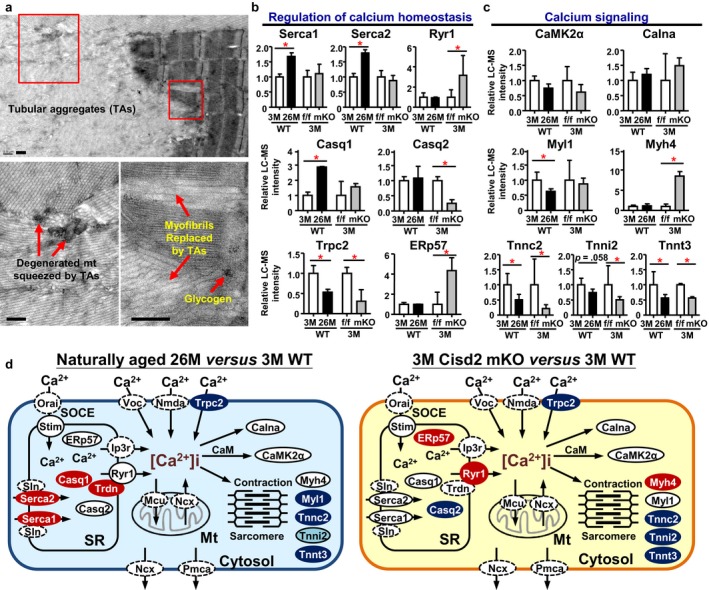
Dysregulation of calcium homeostasis and calcium signaling in naturally and prematurely aged gastrocnemius muscle. (a) Ultrastructural analysis by TEM revealed tubular aggregations (TAs) were present in the gastrocnemius muscles of the naturally aged WT mice at 26M. The red inset boxes in the upper micrograph indicate the magnified area shown in the micrograph below. The presence of TAs occurs only in the old (26M) WT mice and not in the young (3M) WT or the Cisd2 mKO mice. Scale bars, 500 nm. (b) Differential expression of the proteins involved in TAs and regulation of calcium homeostasis. The quantification of each protein is based on the relative intensity of the LC‐MS results from three independent mice of each group. *p <* *.05. (c) Differential expression of the proteins involved in calcium signaling. (d) Schematic illustration of the dysregulation of calcium homeostasis and its potential downstream signaling in the gastrocnemius muscle of naturally aged (26M) WT compared with young (3M) WT mice and prematurely aged Cisd2 mKO compared with WT (Cisd2^f/f^) mice at 3M. Proteins that were not identified in our datasets are circled by dashed lines. Blue indicates down‐regulated, red indicates up‐regulated, and light blue indicates proteins showing a decreasing trend (*p *<* *.1). See Fig. S6c, d for complete protein list

Intriguingly, the TEM analysis of the naturally aged gastrocnemius muscles of WT mice revealed large tubular aggregations (TAs), which seemed to replace myofibrils and squeeze the mitochondria with degenerated morphology (Figure 4a). However, neither 3M Cisd2 mKO nor 3M WT gastrocnemius displayed the TA phenotype. TAs are distinct structures that are composed of closely packed membranous tubules and are considered to be SR‐derived structural abnormalities associated with aging in mice (Agbulut, Destombes, Thiesson & Butler‐Browne, 2000). Although the functional role of TAs and the process by which they develop are not well understood, a previous study has shown that some calcium regulator proteins, including Serca1, Casq1, and RyR1, are components of TAs (Chevessier, Marty, Paturneau‐Jouas, Hantai & Verdiere‐Sahuque, 2004). We therefore analyzed the TA phenotype in relation to the DEPs involved in calcium signaling. Specifically, Casq1 and Serca1 protein levels were found to be significantly increased in the naturally aged gastrocnemius, but not in the prematurely aged gastrocnemius (Figure 4b; Fig. S6a,b). By way of contrast, Ryr1 and Myh4 were significantly increased only in the Cisd2 mKO gastrocnemius, and not in the naturally aged gastrocnemius (Figure 4b,c). Taken together, the dysregulation of calcium homeostasis in the naturally and prematurely aged gastrocnemius muscles appears to be regulated in different ways and to involve different DEPs. A graphical summary comparing the calcium signaling related pathways of the two aging models is presented in Figure 4d.

**Figure 5:**
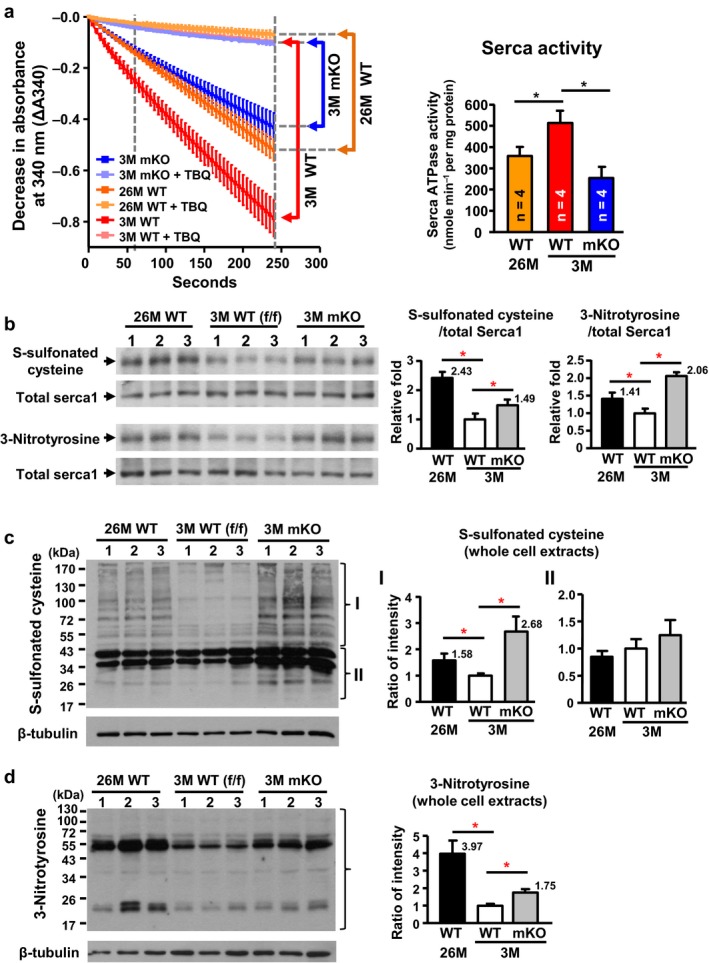
Decreased activity of Serca1 and increased oxidative stress in naturally and prematurely aged gastrocnemius muscle. (a) Significant reductions occurred in calcium‐dependent Serca ATPase activity in naturally aged (26M) WT and prematurely aged (3M) Cisd2 mKO mice. The selective Serca pump inhibitor, TBQ, was used to reflect a specific difference in Serca activity. *n* = 4 for each group of mice. (b) Increased cysteine S‐sulfonation and tyrosine nitration on Serca1 protein in gastrocnemius muscles. The total Serca1 protein was detected by re‐blotting on the same membrane. (c,d) Increase in oxidative modifications, namely S‐sulfonated cysteine and 3‐nitrotyrosine, for all proteins present in whole cell extracts of the gastrocnemius muscle. Quantification of each sample was based on the entire intensities of each lane or region normalized to β‐tubulin. *p < .05

Next, we investigated whether these alterations cause deterioration in muscle contractile function by monitoring the activity of Serca1, which is predominantly expressed and is mainly responsible for the calcium influx into the SR and muscle relaxation in the gastrocnemius (Fig. S7). A pyruvate kinase/lactate dehydrogenase (PK/LDH) coupling reaction was used to investigate the 2,5‐Di‐(tert‐butyl)‐1,4‐benzohydroquinone (TBQ)‐sensitive ATPase activity of Serca using crude homogenates of the gastrocnemius (Gehrig et al., 2012; Simonides & van Hardeveld, 1990). The Serca activity was significantly decreased in naturally aged mice and Cisd2 mKO mice compared with WT young mice (Figure 5a). This would seem to be a surprising discrepancy in relation to the protein levels of Serca, which were found to be up‐regulated in 26M WT mice and at a similar level in 3M Cisd2 mKO mice (Figure 4b). However, previous studies have shown that the activity of Serca is regulated by its redox status. Oxidative modifications of Serca, including cysteine S‐sulfonation and tyrosine nitration, are irreversible modifications that increase with age and inhibit Serca activity (Knyushko, Sharov, Williams, Schoneich & Bigelow, 2005; Qin et al., 2013). To specifically evaluate the oxidative status of Serca1, we blotted the immunoprecipitated Serca1 from gastrocnemius muscle extracts using antibodies against cysteine S‐sulfonation or tyrosine nitration. Importantly, the above oxidative modifications of Serca1 were significantly increased in the Serca1 from naturally and prematurely aged gastrocnemius muscle (Figure 5b), indicating that the impaired activity of Serca is mainly attributable to oxidative modification. We also found that the overall levels of these oxidative modifications of all cellular proteins in the naturally and prematurely aged gastrocnemius muscle were significantly increased (Figure 5c,d).

### 2.5 Dysregulation of energy metabolism, mitochondrial respiration, and ROS response

**Figure 6:**
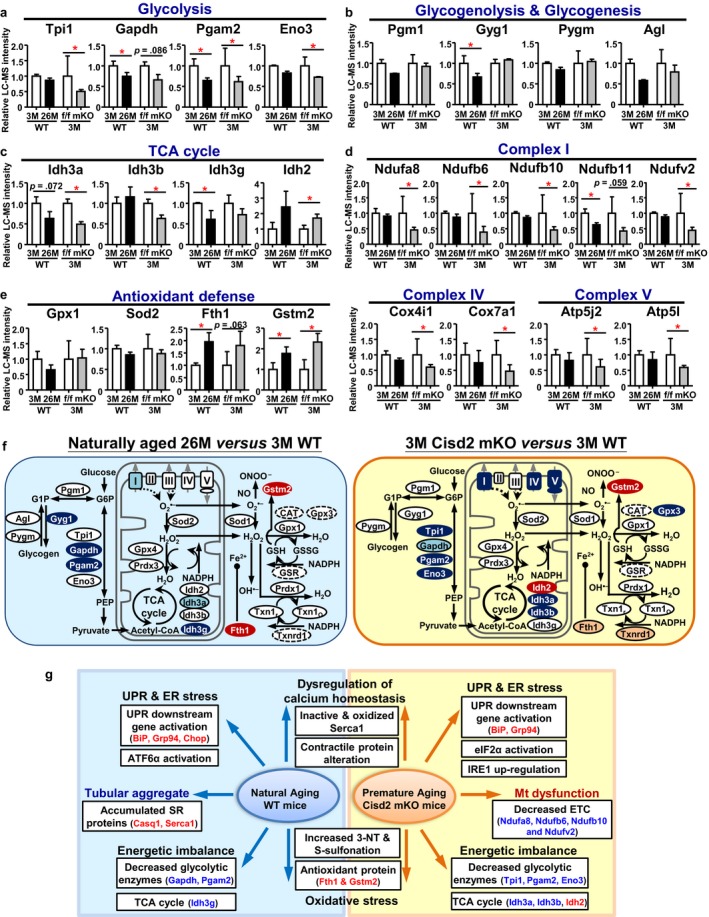
Alterations in energy metabolism and the ROS response in the naturally and prematurely aged gastrocnemius muscle. The differentially expressed proteins involved in the biological pathways of (a) glycolysis, (b) glycogenolysis and glycogenesis, (c) the TCA cycle, (d) complexes I, IV, and V of the electron transport chain, and (e) antioxidant defense. (f) Schematic illustration of the dysregulation of carbohydrate metabolism and the ROS response in the gastrocnemius muscle of naturally aged (26M) WT compared with young (3M) WT mice and prematurely aged Cisd2 mKO compared with WT (Cisd2^f/f^) mice at 3M. Proteins that are not identified in our datasets are circled by dashed lines. Blue indicates down‐regulation and red indicates up‐regulation of DEPs. *p <* *.05. (g) Summary of the comparative proteomics analysis of gastrocnemius muscle in the naturally aged mice and Cisd2 mKO mice. 3‐NT, 3‐nitrotyrosine

Declines in mitochondrial functioning and metabolic homeostasis had been reported previously in aged muscles. For example, ATP content and production have been shown to be decreased by ~50% in the mitochondria isolated from the aged gastrocnemius (Drew et al., 2003). The gastrocnemius contains high levels of glycolytic enzymes and uses glycolysis as its main energy source. Accordingly, the bioenergetic pathways, including glycolysis, glycogen metabolism, mitochondrial electron transport chain (ETC), and tricarboxylic acid (TCA) cycle, were analyzed further. In terms of glycolysis and glycogen metabolism, both the naturally aged mice and Cids2 mKO mice exhibited a tendency toward reduced levels of some enzymes involved in these pathways (Figure 6a,b). In terms of the TCA cycle, most enzymes showed no significant change except the Idh2 and Idh3 (Figure 6c). Remarkably, for mitochondrial ETC, there is a significant decrease in the prematurely aged gastrocnemius of Cisd2 mKO mice of multiple subunits of complex I (Ndufa8, Ndufb6, Ndufb10, and Ndufv2), while there only one subunit of complex I (Ndufb11) was decreased in the naturally aged gastrocnemius (Figure 6d). These findings are consistent with the IPA results wherein the “mitochondrial dysfunction” pathway was significantly enriched in the prematurely aged (Cisd2 mKO vs. Cisd2^f/f^) but not enriched to any great extent in the naturally aged (old vs. young WT) DEP lists (Table S2). Furthermore, given the observation that there is a significant increase in the irreversibly oxidative modifications of all cellular proteins (Figure 5c,d), we analyzed the proteins involved in the antioxidant defense pathways. In the naturally aged mice, Fth1 and Gstm2 were significantly increased, while the Gpx1 and Sod2 showed a trend toward a decrease. In the Cisd2 mKO mice, Fth1 and Gstm2 were also increased (Figure 6e). A graphical summary comparing energy metabolism and the ROS response pathways between the two mouse models is presented in Figure 6f.

## 3 DISCUSSION

Four new findings are identified in this study. Firstly, there is a significant down‐regulation of Cisd2 protein (~30% remains) in the gastrocnemius of middle‐aged and old WT mice, suggesting that Cisd2 may play a crucial role in gastrocnemius aging. Secondly, Cisd2 mKO mice have an overt phenotype that leads to degeneration of skeletal muscles, destruction of mitochondria, and destruction of ER/SR, together with impairment of proteostasis. Importantly, these phenotypic characteristics are very similar to those observed in naturally aged mice regarding pathological alterations, ultrastructural abnormalities, and proteomic profiling. Thirdly, in addition to common protein changes, there are unique pathway alterations and subcellular changes involved in the natural and premature aging processes. In particular, TAs, which are associated with dysregulation of calcium homeostasis, were detected only in naturally aged mice. In contrast, while the “mitochondrial dysfunction” pathway was significantly enriched in Cisd2 mKO mice, this only occurred to a lesser extent in naturally aged mice. Finally, the redox status of Serca1 is significantly altered and there is oxidative modification of Serca1 that obviously damages its functionality leading to an impaired calcium‐dependent Serca activity and a consequential defect in calcium uptake by the ER/SR. Many ER chaperones are calcium‐dependent for optimal activity, and therefore, a reduction in the ER calcium will reduce chaperone function and ER folding capacity, thereby cause ER stress and turn on UPR signaling. These findings provide an explanation, at least in part, as to why the pathways for calcium signaling and UPR/ER stress stand out above the rest of the age‐related pathway changes. Moreover, the up‐regulation of Serca protein in the naturally aged gastrocnemius might be a compensation in response to damaged Serca protein, which would lead to functional loss.

In this study, we provided evidence for the first time to substantiate that the idea that Cisd2 plays a crucial role in the skeletal muscles and that it functions in a cell‐autonomous manner to maintain the integrity of skeletal muscles. In middle‐aged WT mice, the dramatic reduction in Cisd2 in the gastrocnemius correlated with the presence of obvious pathological and biochemical alterations (Fig. S8). Interestingly, in the soleus of WT mice, the Cisd2 level is maintained consistently during aging (Fig. S9a,b), and this correlates well with the observation that no overt degeneration of the soleus was found at old age (Fig. S9c). However, in the Cisd2 mKO mice, both the soleus and gastrocnemius displayed an overt degeneration phenotype (Fig. S9d,e), suggesting that Cisd2 also plays a crucial role in the soleus and that a consistent level of Cisd2 in the soleus of WT mice may help preserve its mass and function during natural aging.

The tissue‐specific Cre recombinase used in this study was driven by the MCK promoter. This begins to express at an embryonically late stage, namely E17 just before birth, and reaches maximal levels at postnatal day 10, remaining at a constantly high‐level throughout the rest of mouse's life (Bruning et al., 1998). Therefore, the phenotypic effect of Cisd2 knockout of skeletal muscle would seem to occur after the postnatal stage rather than during prenatal early development. Regarding the natural aging of WT mice, 18 of the 46 DEPs have also been identified by other groups as involved in aging, including P4hb (McDonagh, Sakellariou, Smith, Brownridge & Jackson, 2014), Casq1 (McDonagh et al., 2014), Pgam2 (Chaves et al., 2013), and Gstm2 (Chaves et al., 2013), suggesting common protein alterations can be found by different experimental approaches.

In humans, TAs have been reported to appear in the skeletal muscles during a variety of disorders including various neuromuscular disorders and TA myopathy. The latter is a rare muscle disease associated with muscle weakness and cramps in humans (Chevessier et al., 2005). In mouse models, the structure of TAs can be experimentally induced by anoxic conditions (Schiaffino, Severin, Cantini & Sartore, 1977). The development of TAs has been associated with several factors, including age (beginning at middle age), sex (usually male) and fiber type (type II fast‐twitch) in WT mice (Agbulut et al., 2000). Notably, TA structures were not observed either in the gastrocnemius of young WT male mice or in the gastrocnemius of prematurely aged Cisd2 mKO male mice at 3 months of age. These findings may be attributable to the age of the mice as the formation of TAs appears to be a time‐dependent process requiring a long incubation time to allow for the gradual formation of TAs (Lahoute et al., 2008; Nishikawa et al., 2000; Zhou et al., 2013). Furthermore, a previous study has shown that Casq and Serca both participate in TA development. As TAs appear to be associated with myopathy, Serca located in the TA membrane has been suggested to be inactivated or functionally compromised (Boncompagni, Protasi & Franzini‐Armstrong, 2012). Consistent with the above, our current findings in mice, and previous findings using a rat model (Sharov, Dremina, Galeva, Williams & Schoneich, 2006), have both revealed that Serca activity decreases in an age‐dependent manner and this decrease in Serca activity is correlated inversely with increased oxidative modification, namely cysteine sulfonation and 3‐nitrotyrosine, which occurs as a consequence of oxidative stress and aging.

If we consider the “mitochondrial dysfunction” pathway, this was significantly enriched in the prematurely aged gastrocnemius of Cisd2 mKO mice, and this also occurred, but only to a lesser extent, in the naturally aged mice. It should be noted that these DEPs are involved in oxidative phosphorylation within the mitochondria and are all encoded by nuclear genes. Accordingly, in addition to the presence of damaged mtDNA in the naturally aged muscle (Wu et al., 2012), the functional decline affecting mitochondria may also be attributable to the impaired protein synthesis of nuclear‐encoded mitochondrial proteins. Damage to mitochondrial proteins and mtDNA is known to promote ROS generation, and this forms a vicious cycle that increasingly damages cellular molecules. The iron‐sulfur cluster‐bearing CDGSH domain is the only one functional domain known within Cisd2, and Cisd2 knockout is likely to lead to oxidative stress due to impaired redox activity and/or disrupted iron homeostasis. Fth1 and Gstm2 are involved in the anti‐oxidation reactions necessary to resolve oxidative stress, and they do this via sequestration of free ions and detoxification of reactive aldehydes, respectively. Interestingly, the protein levels of Fth1 and Gstm2 were significantly increased (Figure 6e) in the gastrocnemius of the Cisd2 mKO mice and also in the naturally aged mice, where there is a decrease of ~70% in Cisd2 protein, with about 30% remaining, compared with young mice. These two changes may be linked.

## 4 EXPERIMENTAL PROCEDURES

### 4.1 Mice

Cisd2 floxed allele (Cisd2^f/f^) transgenic mice were generated as previously described (Wang, Chen, et al., 2014). The muscle creatine kinase‐Cre transgenic (MCK‐Cre) mice were purchased from the Jackson Laboratory (JAX 006475) and bred with Cisd2^f/f^ mice to generate muscle‐specific Cisd2 knockout (mKO) mice. All mice analyzed were male with a C57BL/6 background. The mice were bred in a specific pathogen‐free facility. The Institutional Animal Care and Use Committees (IACUC) of the National Yang‐Ming University approved this study.

### 4.2 Pathological analysis

Mouse gastrocnemius muscles were collected, fixed with 10% formalin, and then embedded in paraffin. Standard hematoxylin and eosin (H&E) staining of tissue sections (3–4 μm) was carried out. The ultrastructure of the mouse gastrocnemius muscles was examined by transmission electron microscopy (TEM) (Kao, Chen, Kuo & Yang, 1995).

### 4.3 Preparation of mouse skeletal muscle proteome

Frozen mouse tissue samples were subjected to homogenization using RIPA buffer containing protease inhibitors. Next, the homogenate was centrifuged at 13,000 *g* (20 min) twice to pellet insoluble cell debris. The final supernatant was collected and quantified.

### 4.4 Quantitative proteomics analysis

The protein samples were resolved by SDS‐PAGE and excised into five fractions per lane for tryptic digestion. The extracted peptides were analyzed by LTQ‐Orbitrap hybrid tandem mass spectrometer (Thermo Fisher). The raw data were processed by PEAKS software version 7.5 for protein identification and label‐free quantification (Zhang et al., 2012). The DEP list was obtained using a 1.5‐fold cutoff with a significance threshold of *p *<* *.05. The procedure is described in Methods S1 section.

### 4.5 Pathway analysis

The DEPs were further annotated using Gene Ontology via the PANTHER online tools (www.pantherdb.org) or analyzed by the IPA approach (Ingenuity Systems®, www.ingenuity.com). Heat maps were created of proteins present in significant canonical pathways by loading log‐transformed fold changes into Multi Experiment Viewer (MEV) 4.9 software (Saeed et al., 2003).

### 4.6 RNA analysis

Total RNA was isolated from muscle using TRIzol reagent (Life Technology). Reverse transcription and real‐time quantitative PCR were conducted as previous described (Lin et al., 2012).

### 4.7 Western blotting

The following antibodies were used for Western blotting: Cisd2 (Chen et al., 2009); β‐tubulin (05‐661; Upstate); ATF‐6α (IMG‐273; Imgenex); eIF2α (#9722; Cell Signaling); p‐eIF2α (Ser51, #3398; Cell Signaling); IRE1α (#3294; Cell Signaling); p‐IRE1α (Ser724, PA1‐16927; Thermo); 3‐Nitrotyrosine (ab61392; Abcam); Cysteine (sulfonate) (ADI‐OSA‐820; Enzo); and Serca1 (MA3‐912; Thermo). Quantitative densitometric analysis was performed using ImageJ software.

### 4.8 Sarco/endoplasmic reticulum calcium‐ATPase (Serca) activity assay

SR calcium‐ATPase activity levels were assayed using an enzyme‐coupled spectrophotometric assay as described previously (Gehrig et al., 2012; Simonides & van Hardeveld, 1990). See Methods S1.

### 4.9 Immunoprecipitation and modification of Serca1

α‐Serca1 was incubated with protein A Mag Sepharose Xtra beads (GE Healthcare Life Sciences) at 4°C for 4 hr; these were then incubated with gastrocnemius lysate at 4°C overnight. After washing with 0.1% Triton X‐100/PBS buffer, the Serca1 proteins were eluted and analyzed by immunoblotting to measure oxidative modifications.

### 4.10 Statistical analysis

The results are presented as mean ± SD from at least three independent experiments. Comparisons between two groups were carried out using PEAKS software for quantitative proteomics and Student's *t* test for other quantification. A *p‐*value of less than .05 was considered significant.

## CONFLICT OF INTEREST

None declared.

## AUTHOR CONTRIBUTIONS

YLH performed the proteomics experiments, analyzed the results, and drafted the manuscript; ZQS performed Western blot analyses and the Serca experiments; CYW established the Cisd2 mKO model and performed its initial characterization; YCT participated in the pathological analysis; CCL participated in the proteomics experiments; CHK performed TEM and analyzed the results; LKC participated in conceptual discussion and experimental design; CHL and TFT designed the experiments, analyzed the results, interpreted the findings, and wrote the final manuscript.
