# Supplementary material for: Comparative proteomic profiling reveals a role for Cisd2 in skeletal muscle aging
Source: Aging Cell. 2017 Nov 23;17(1):e12705. doi: 10.1111/acel.12705 (PMC5770874; doi:10.1111/acel.12705)
Supplement: Supplementary file 1 [file ACEL-17-na-s001.pdf]

Supplementary Methods

Serca activity assay

The gastrocnemius muscle was homogenized with the ice-cold homogenization buffer (250 mM sucrose, 5 mM HEPES, 0.2% NaN_3 and protease inhibitor cocktail at pH 7.3). The muscle homogenate was allowed to react with 1 μM calcimycin A-23187 and 1 mM $CaCl_2$ at a protein concentration of 50 μg/mL in 1 mL of reaction buffer (200 mM KCl, 20 mM HEPES, 15 mM $MgCl_2$, 10 mM NaN_3, 10 mM Phosphoenolpyruvate, 5 mM ATP, 1 mM EGTA at pH 7.3) for 3 min at 37°C. The reaction mixture was further added with 18 U/mL lactate dehydrogenase (LDH), 12 U/mL pyruvate kinase (PK), and 0.5 mM NADH, and then loaded into a plastic cuvette immediately before measurement. The absorbance of 340 nm at 37°C was recorded using a UV/visible spectrophotometer. Serca-dependent Ca^{2+}-ATPase activity was determined in the presence versus abscence of the Serca-specific inhibitor 2',5'-di(tert-butyl)-1,4-benzohydroquinone (TBQ) (40μM). Measurement of oxidized NADH (340 nm) level was peformed through calculating the absorption coefficient of NADH (6.22 mM^{-1} x cm^{-1}). The activity of ATPase was determined by normalizing oxidized NADH level (nmole) to reaction time (min) and protein amount (mg). Activity (right panel) was calculated based on A340 recorded from 60 to 240 seconds (Δ60-240s) showing as dashlines of the left panel in Figure 5a.

Protein separation and in-gel digestion

Protein lysates were dissolved in sample buffer (2% SDS, 5% β-mercaptoethanol, 10% glycerol and 50mM Tris-HCl, pH 6.8) and heated to 95°C for 10 min. 40μg of protein lysates was resolved by 10% SDS-PAGE and the resulting gel was stained with Colloidal Coomassie brilliant blue G250 for overnight. Each protein lane in gel was equally cut into 5 gel segments. Each segment was further sliced into pieces and transferred into a 1.5ml eppendorf tube (supplementary material, Fig. S2a). These gel pieces was destained and dehydrated with 50% acetonitrile (ACN) /25mM ammonium bicarbonate and followed by washing with 100% ACN. In-gel digestion of gel pieces was performed with sequencing-grade modified trypsin (Promega) at 37°C for 20 hours. The resulting tryptic peptides were extracted from gel pieces through sonication twice in 50% ACN in 0.1% trifluoroacetic acid aqueous solution.

Liquid chromatography–tandem mass spectrometry (LC-MS/MS)

The extracted tryptic peptides were dried and re-dissolved in 0.1% formic acid aqueous solution for the subsequent analysis using LTQ-Orbitrap hybrid tandem mass spectrometer (Thermo Fisher). The mass spectrometer was inline coupled with nanoflow HPLC system (Agilent) equipped with a C18 trap column (5 mm length, 300 μm i.d. and 5 μm beads, PepMap 100, Thermo) and a self-packed C18 separation column (10 cm length, 75 μm i.d. and 5 μm beads). Gradient elution of peptides in nanoLC was from 2 to 35% ACN in a 0.1% formic acid aqueous solution at 800 nL/min flow rate for 85 min. In addition to the full scan mass spectra (MS1) recording all detecable peptide mass, the MS/MS spectra ranging from 200 to 1500 m/z were acquired in a data-dependent analysis (DDA) mode with the Top5 abundance method.

Data analysis

All raw MS data were further processed by PEAKS software version 7.5. With Peaks DB module for peptide identification, the intepreted data were searched against mouse Swiss-Prot database (version 2015-02) with a precursor mass tolerance set to 20 ppm and a fragment ion mass tolerance set to 0.8 Da. In addition, only one trypsin missed cleavage sites was allowed and variable modification was limited to methionine oxidation. The FDR for identified peptide-spectrum matches was set to 1% for each dataset. At least one significant unique peptide per protein was required for positive identification. A label-free quantification module was further applied to semi-quantify the protein abundance with the following parameters: a mass error tolerance of 40 ppm and a retention time shift tolerance of 6 min. Differentially expressed proteins (DEPs) were definied based on their abundance with fold change > 1.5 along with significance (-10 log *p*) > 13 (equivalent to a *p*-value of 0.05) when compared to control. In addition, at least two unique peptides were identified from at least two out of six MS analysis for DEP.

Western Blot

Protein lysates from mouse muscle tissues were separated by SDS-PAGE (Bio-Rad) and then electro-transferred to a polyvinylidene fluoride membrane. Upon blocking with 5% (w/v) nonfat dry milk or 5% BSA, the resulting membranes were incubated with the corresponding primary antibody overnight at 4°C. Subsequent incubation of such membrane with appropriate secondary antibody which couples with horseradish peroxidase was performed. After three times wash with Tris-buffered saline/Tween 20 (TBST), the membrane was subject to the detectetion of chemiluminescence using peroxidase-specific substrates (Millipore).

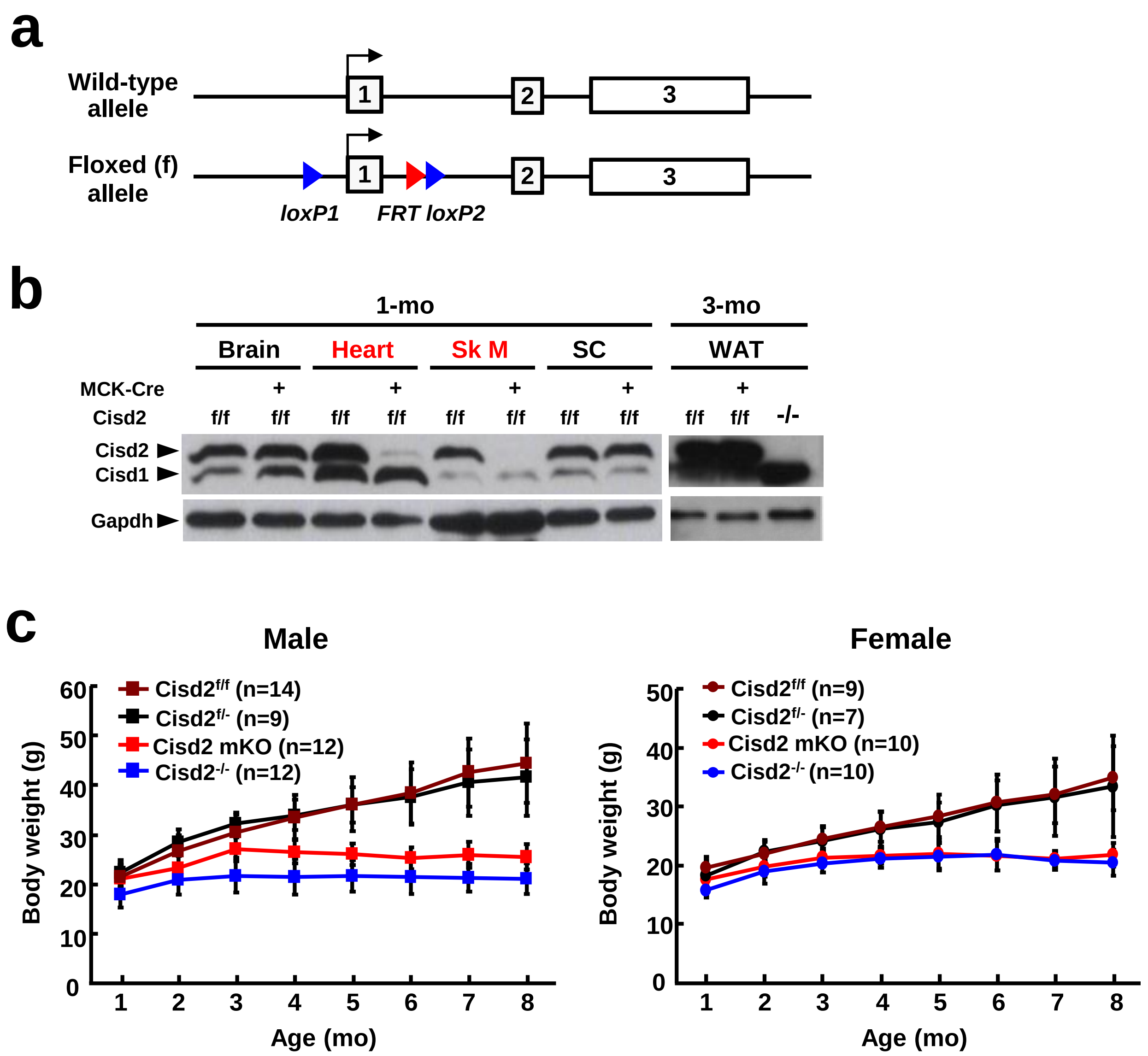

Fig. S1 Generation of muscle-specific Cisd2 KO (Cisd2 mKO) mice by breeding of Cisd2$^{f/f}$ and MCK-Cre mice. **(a)** Structure of Cisd2 floxed (Cisd2$^{f/f}$) allele and WT allele. **(b)** Cisd2 protein expression of different tissues in Cisd2 mKO mice. Sk M, skeletal muscle; SC, spinal cord; WAT, white adipose tissue. **(c)** Growth curve of male and female mice for the Cisd2$^{f/f}$ (control), Cisd2$^{f/-}$ (heterozygous KO), Cisd2 mKO and Cisd2$^{-/-}$ (conventional homozygous KO) groups of mice.

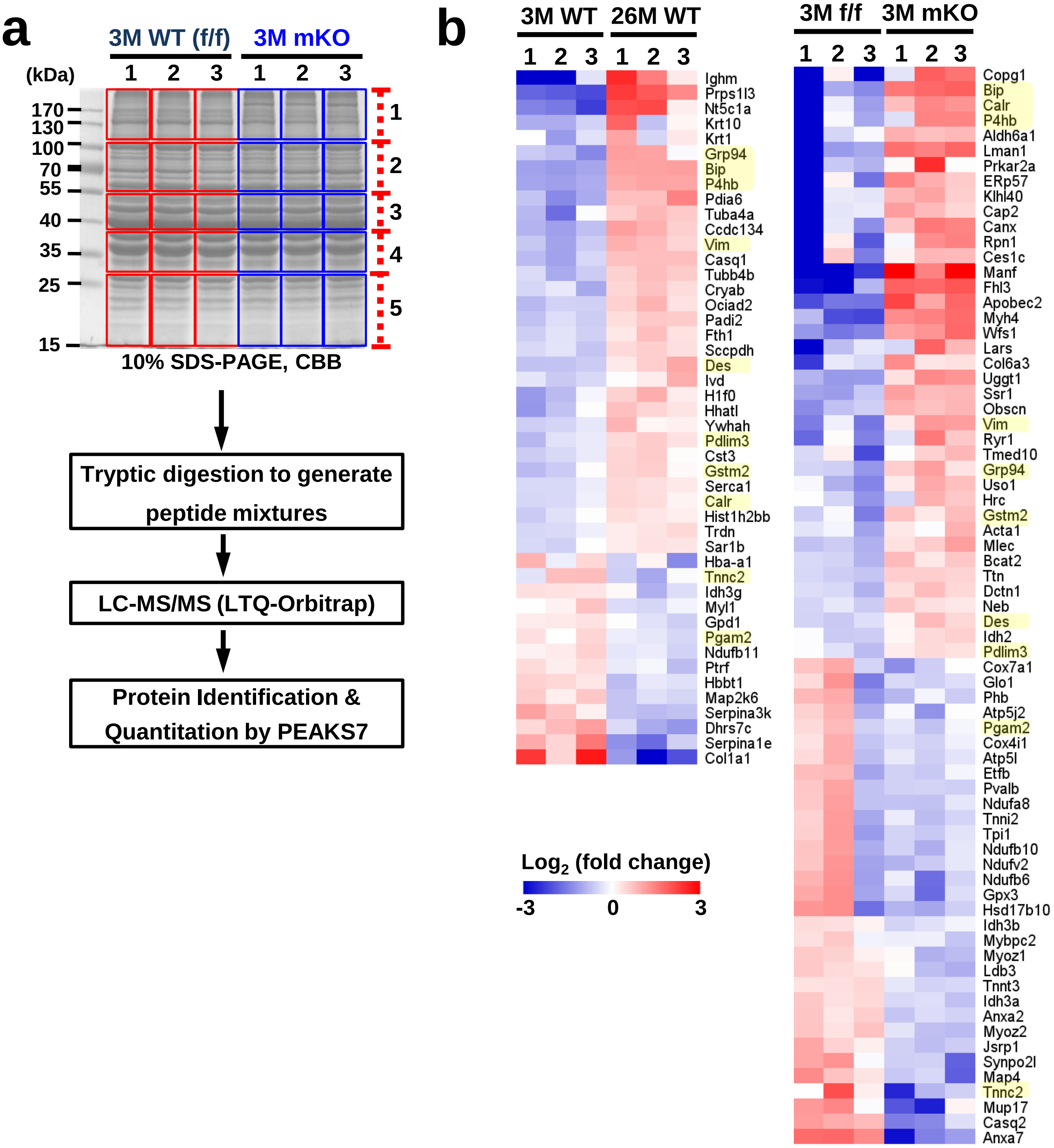

Fig. S2 Summary of proteomics method and whole DEP list identified by PEAKS 7.5. **(a)** Schematic diagram of a label-free proteomics approach **(b)** Heat maps of DEPs in the gastrocnemius of two aging models. Red indicates up-regulation and blue indicates down-regulation. Yellow marks the ten DEPs identified in both datasets.

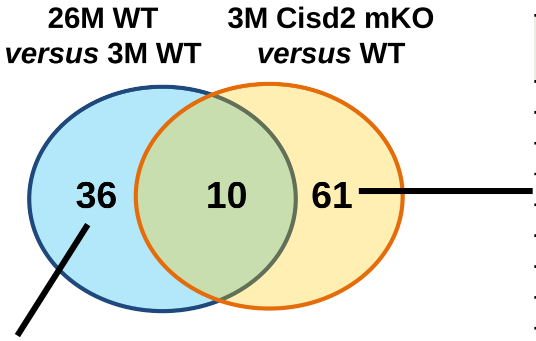

No.	Gene	Significance (-10 log *p*)	old/yg
1	Prps1l3	71.30	**14.73**
2	Nt5c1a	68.17	**10.96**
3	Ighm	48.20	**10.73**
4	Pdia6	39.57	**4.25**
5	Ccdc134	51.95	**3.52**
6	Krt10	68.77	**3.05**
7	Casq1	113.37	**2.89**
8	Tuba4a	18.37	**2.63**
9	Tubb4b	13.35	**2.48**
10	Ociad2	16.92	**2.38**
11	H1f0	17.24	**2.33**
12	Padi2	18.99	**2.17**
13	Cryab	46.56	**2.15**
14	Hhatl	56.15	**2.13**
15	Fth1	34.58	**1.95**
16	Sccpdh	15.43	**1.94**
17	Ivd	24.78	**1.88**
18	Ywhah	15.20	**1.85**
19	Krt1	45.32	**1.81**
20	Cst3	21.18	**1.80**
21	Serca1	29.77	**1.67**
22	Trdn	27.53	**1.63**
23	Hist1h2bb	19.26	**1.53**
24	Sar1b	13.95	**1.51**
25	Gpd1	14.46	**0.67**
26	Myl1	16.05	**0.63**
27	Ndufb11	17.59	**0.63**
28	Ptrf	15.42	**0.62**
29	Idh3g	20.07	**0.61**
30	Hbbt1	46.99	**0.54**
31	Hba-a1	38.71	**0.52**
32	Map2k6	15.20	**0.49**
33	Serpina3k	41.60	**0.37**
34	Dhrs7c	25.35	**0.31**
35	Serpina1e	26.40	**0.21**
36	Col1a1	20.88	**0.05**

No.	Gene	Significance (-10 log *p*)	mKO/WT
1	Manf	15.29	**57.62**
2	Fhl3	35.85	**13.64**
3	Apobec2	50.64	**11.45**
4	Lman1	22.73	**10.21**
5	Myh4	88.71	**8.50**
6	Wfs1	14.24	**7.04**
7	Copg1	17.22	**6.71**
8	Prkar2a	27.53	**6.45**
9	Canx	40.04	**5.97**
10	Lars	14.96	**5.15**
11	Uggt1	14.88	**4.39**
12	Rpn1	26.09	**4.35**
13	Pdia3	22.34	**4.33**
14	Ssr1	40.44	**3.67**
15	Klhl40	26.48	**3.30**
16	Cap2	24.42	**3.28**
17	Aldh6a1	18.78	**3.23**
18	Obscn	14.13	**3.21**
19	Ryr1	14.45	**3.16**
20	Uso1	15.38	**3.00**
21	Col6a3	18.78	**2.88**
22	Mlec	15.18	**2.80**
23	Ces1c	14.26	**2.46**
24	Bcat2	13.87	**2.32**
25	Ttn	17.18	**2.21**
26	Dctn1	19.74	**2.19**
27	Tmed10	16.41	**2.02**
28	Hrc	28.86	**1.96**
29	Neb	13.2	**1.88**
30	Acta1	23.86	**1.87**
31	Idh2	14.14	**1.71**
32	Mybpc2	19.92	**0.63**
33	Idh3b	17.22	**0.63**
34	Atp5j2	13.25	**0.61**
35	Cox4i1	15.48	**0.60**
36	Atp5l	13.7	**0.59**
37	Tnnt3	16.65	**0.56**
38	Etfb	20.32	**0.56**
39	Glo1	13.11	**0.53**
40	Myoz1	18.16	**0.52**
41	Tnni2	15.07	**0.51**
42	Ldb3	24.14	**0.51**
43	Tpi1	20.21	**0.51**
44	Phb	13.05	**0.51**
45	Idh3a	18.12	**0.49**
46	Anxa2	18.13	**0.48**
47	Pvalb	32.14	**0.48**
48	Cox7a1	15.1	**0.47**
49	Ndufa8	13.15	**0.46**
50	Ndufb10	16.82	**0.46**
51	Ndufv2	20.74	**0.45**
52	Myoz2	16.31	**0.43**
53	Jsrp1	17.57	**0.41**
54	Ndufb6	18.19	**0.39**
55	Gpx3	14.99	**0.36**
56	Hsd17b10	14.66	**0.33**
57	Synpo2l	23.11	**0.30**
58	Map4	14.5	**0.29**
59	Mup17	15.12	**0.25**
60	Casq2	14.43	**0.23**
61	Anxa7	19.71	**0.10**

***p*-value < .05, -10 log *p* > 13**

Fig. S3 List of 36 and 61 DEPs uniquely identified in naturally aged (old WT *vs.* young WT) and prematurely degenerated (3M Cisd2 mKO vs. Cisd2$^{f/f}$) gastrocnemii, respectively.

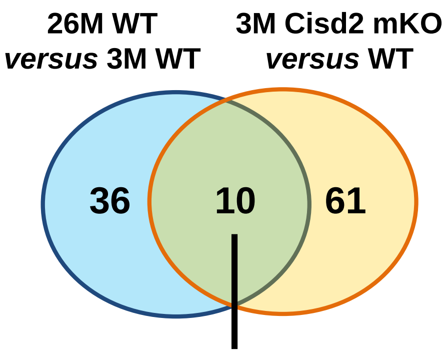

No.	Gene	Protein name	old/yg	Significance (old *vs.* yg)	mKO/WT	Significance (mKO *vs.* WT)
1	Hspa5	78 kDa glucose-regulated protein (Grp78), BiP	**4.65**	78.56	**10.19**	87.38
2	P4hb	Prolyl 4-hydroxylase, beta polypeptide	**4.27**	94.57	**4.36**	41.15
3	Hsp90b1	Heat shock protein 90 beta (Grp94) member 1, endoplasmin	**3.48**	24.33	**2.74**	26.29
4	Vim	Vimentin	**2.89**	16.63	**3.67**	28.04
5	Des	Desmin	**2.53**	68.6	**2.02**	19.76
6	Pdlim3	PDZ and LIM domain protein 3	**1.95**	20.77	**1.64**	26.69
7	Gstm2	Glutathione S-transferase Mu 2	**1.78**	15.72	**2.32**	21.98
8	Calr	Calreticulin	**1.64**	13.79	**4.58**	45.41
9	Pgam2	Phosphoglycerate mutase 2	**0.65**	19.17	**0.61**	23.93
10	Tnnc2	Troponin C, skeletal muscle	**0.51**	17.82	**0.22**	21.54

Fig. S4 List of the ten DEPs identified in both datasets.

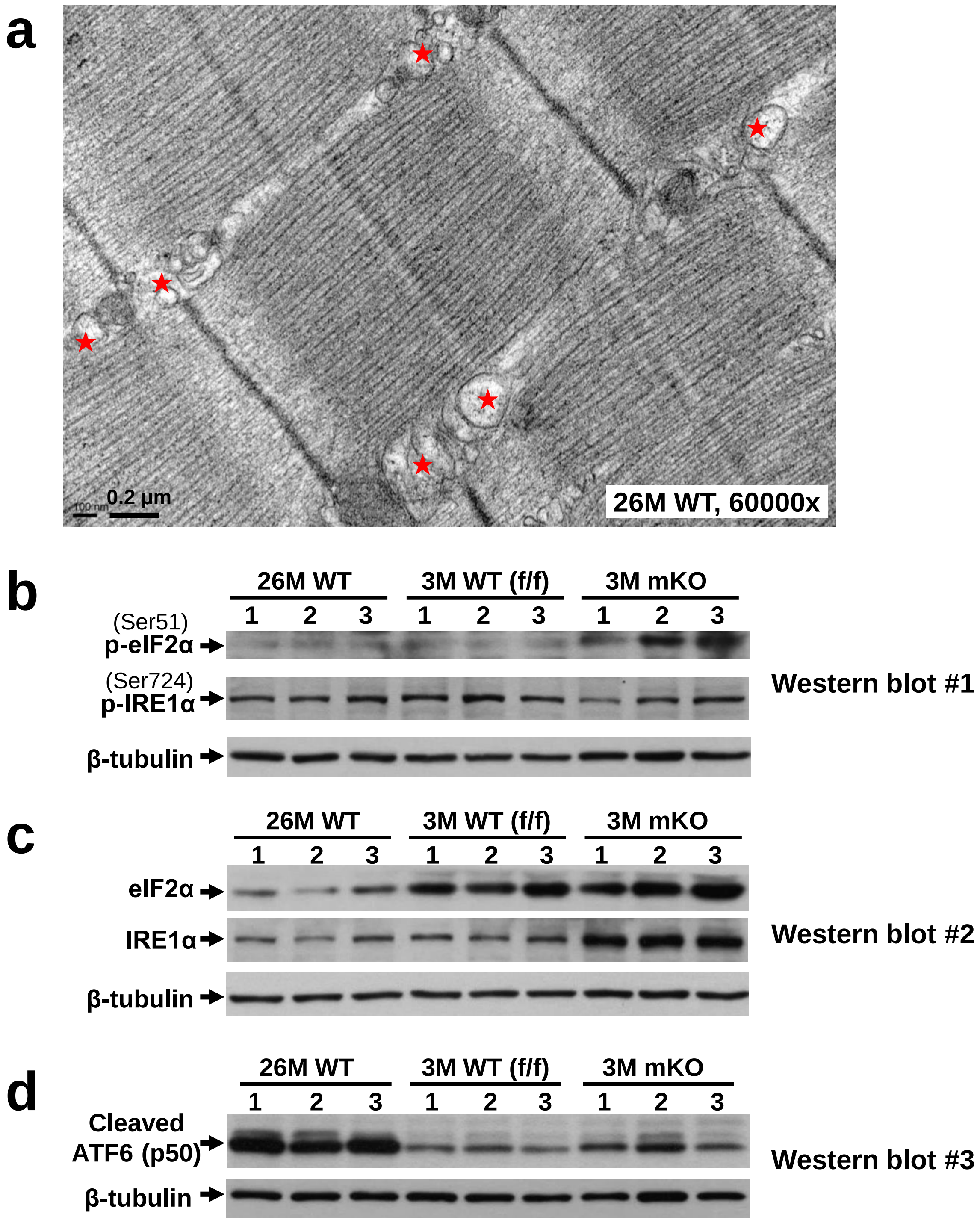

Fig. S5 Ultrastructural abnormality of naturally aged gastrocnemius of WT mice, and Western blots of β-tubulin for the normalization of protein loading of the three UPR pathways. **(a)** Remarkable SR dilation and degeneration (red stars) in the gastrocnemius muscles of 26M WT mice were revealed by TEM. **(b-d)** Three Western blots were prepared for quantification of the phosphorylated form of p-eIF2α & p-IRE1α (blot #1), total proteins of eIF2α & IRE1α (blot #2), and cleaved ATF6 (blot #3). The β-tubulin was used as an internal control for protein loading for each Western blot.

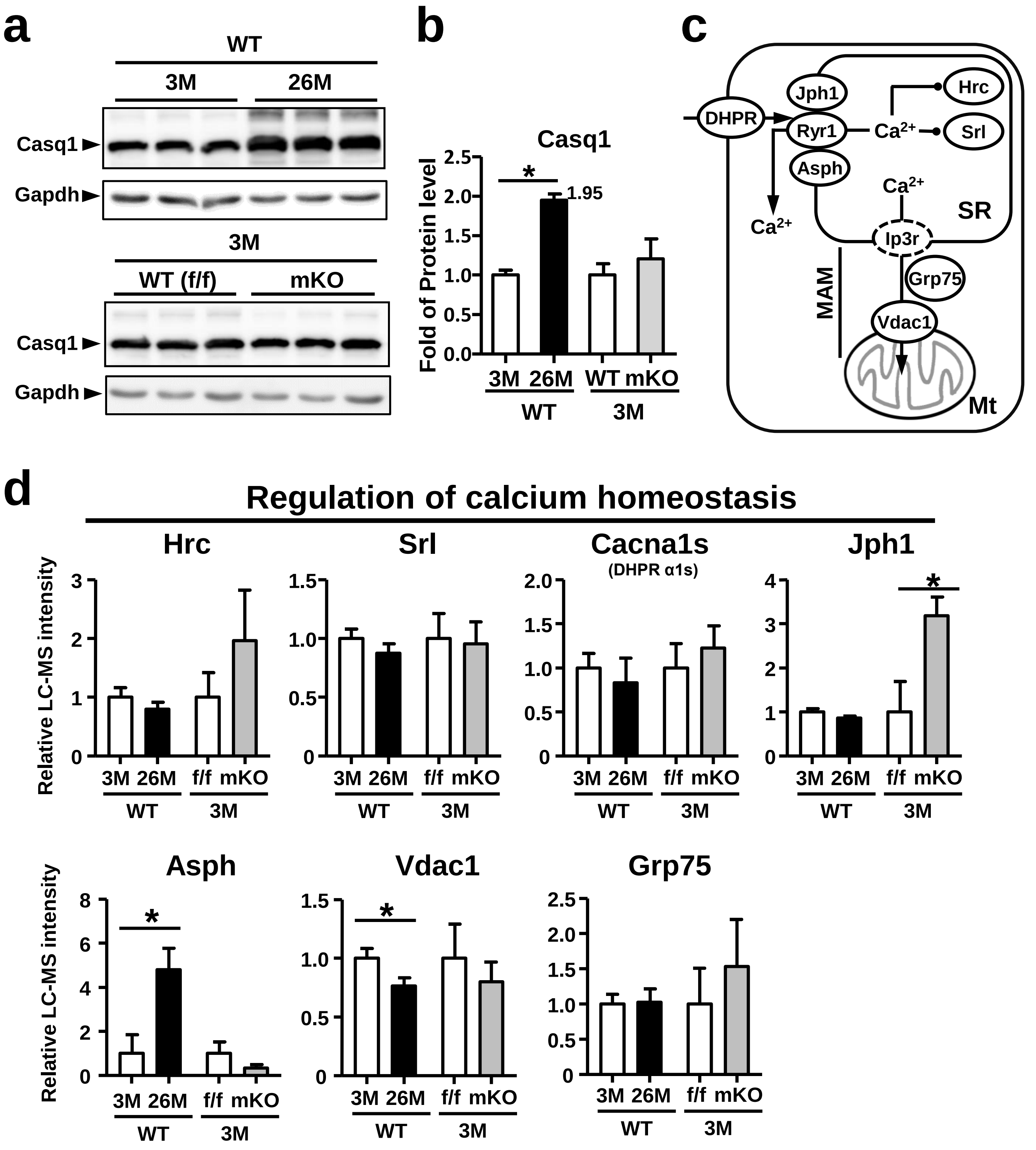

Fig. S6 Supplementary results to the dysregulation of calcium homeostasis and calcium signalling presented in Figure 4. **(a-b)** Immunoblot analysis of Casq1 in gastrocnemius of naturally aged and Cisd2 mKO mice. N=3 for each group. **(c-d)** Differential expression of proteins involved in regulation of calcium homeostasis.

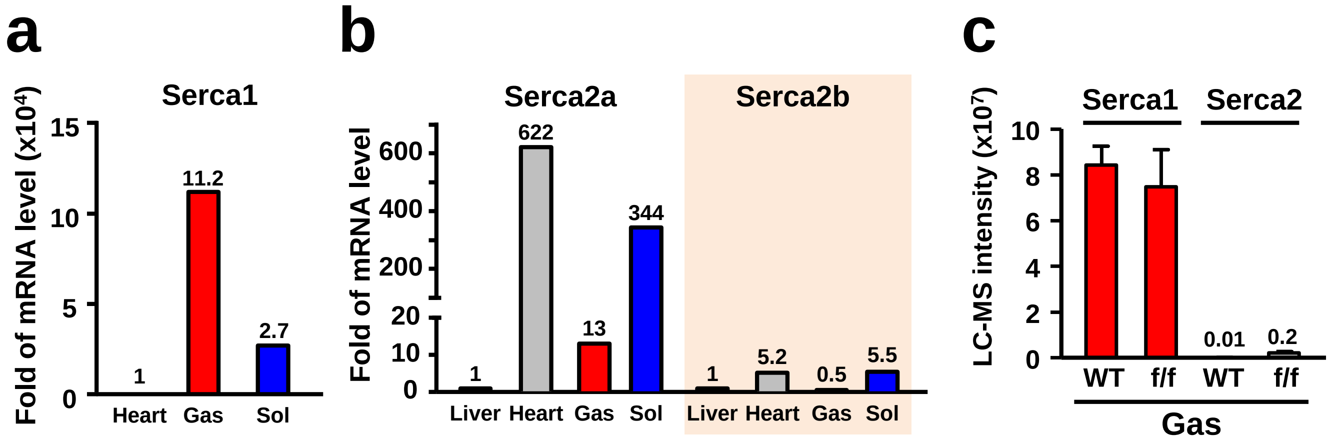

Fig. S7 Quantification of Serca1, Serca2a and Serca2b by real time RT-qPCR (a, b) and LC-MS/MS (c) using tissues obtained from 3-month old WT male mice. **(a)** Serca1 is the major Serca isoform expressed in the gastrocnemius (Gas). **(b)** Quantification of Serca2a and Serca2b in the liver, heart, gastrocnemius (Gas) and soleus (Sol). The mRNA expression levels were normalized to *Hprt*. **(c)** MS-based protein quantification of Serca1 and Serca2 in the gastrocnemius of 3M WT and Cisd2$^{f/f}$ mice.

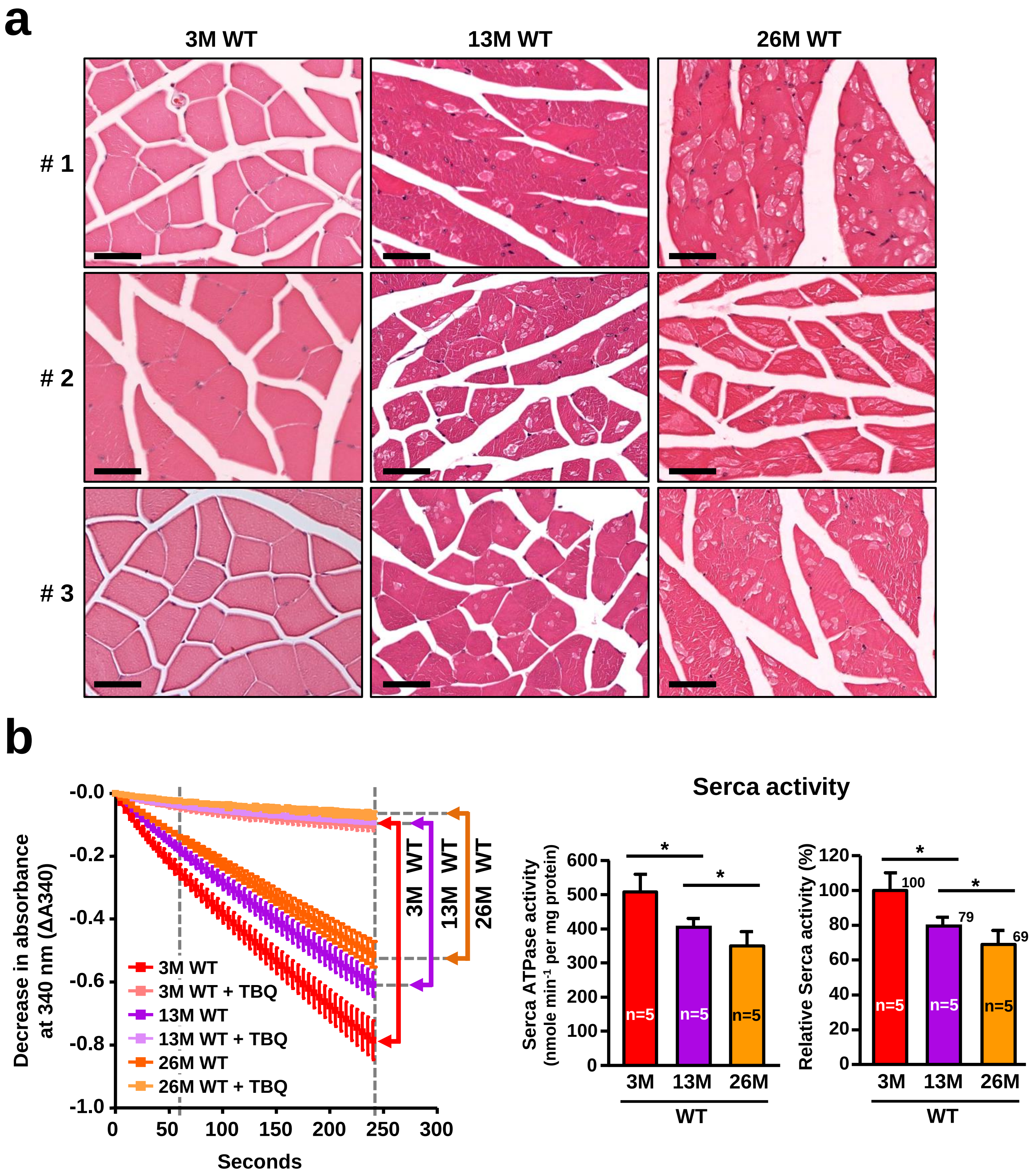

Fig. S8 Age-related degeneration and impaired Serca activity in the gastrocnemius of WT mice. **(a)** H&E staining of the gastrocnemius muscle of 3M, 13M and 26M WT mice. N=3 mice for each group. Scale bars, 50 μm. **(b)** Age-dependent reduction of calcium-dependent Serca ATPase activity in the gastrocnemius at middle age (13M) and old age (26M) compared with young (3M) WT mice.

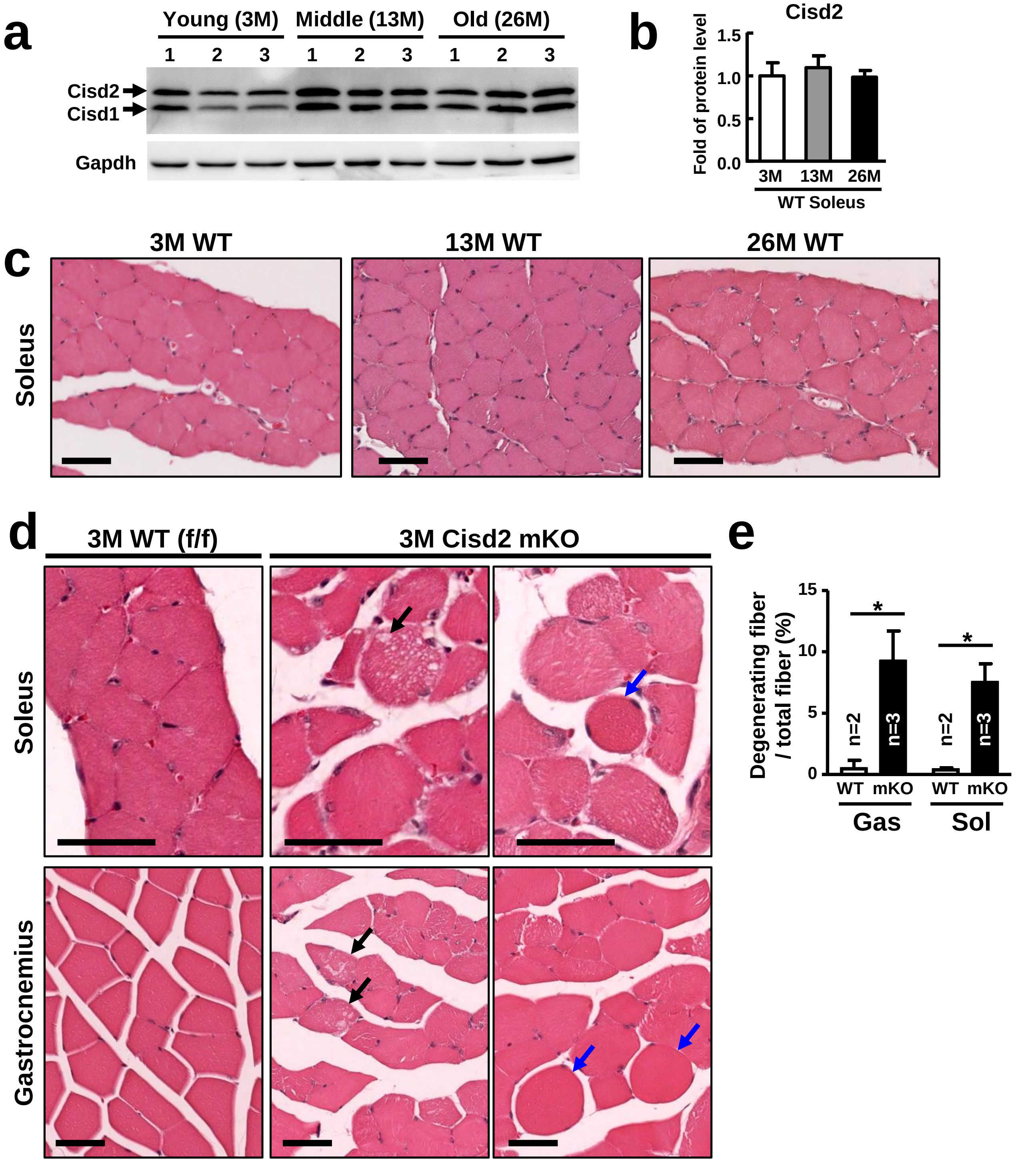

Fig. S9 Expression levels of Cisd2 protein in the soleus of WT mice and histopathological analysis of the soleus of naturally aged mice and Cisd2 mKO mice. **(a) (b)** Cisd2 protein levels in the soleus at middle age (13M) and old age (26M) compared with young (3M) WT mice. The protein expression levels were normalized to Gapdh. **(c)** H&E staining of the soleus of 3M, 13M and 26M WT mice. Scale bars, 50 μm. **(d)** H&E staining of the soleus and gastrocnemius muscles of 3M WT (f/f) and 3M Cisd2 mKO mice. The black arrows indicated degenerative loss of muscle fibers. The blue arrows indicate the rounded fibers. Scale bars: upper and lower panels, 50 μm. **(e)** Percentage of degenerating (rounded or vacuolated) fibers was significantly increased in the soleus and gastrocnemius muscles of Cisd2 mKO mice. Three microscopic fields (200X) were examined and at least 200 myofibers were analyzed for each mice.

Table S1 Summary of total number of identified proteins and differentially expressed proteins (DEPs) by PEAKS label-free quantification software.

Group (each n=3)	Total identified proteins (1% FDR)	Proteins for quantification[a]	Differentially expressed proteins (DEPs)[b]
26-mo WT *versus* 3-mo WT	865	784	46 (32 up / 14 down)
3-mo Cisd2 mKO *versus* 3-mo WT	1021	1000	71 (39 up /32 down)

[a], proteins detected in at least 2 of 6 samples with at least 1 unique peptides; [b], $p < .05$ & fold change >1.5 with at least 2 unique peptides

Table S2 Ingenuity canonical pathways list in naturally aged and Cisd2 mKO mice. The order is based on *p*-value of overlap between pathway molecules and our datasets. Red marks the pathway with *p*-value < .05 and yellow marks the functional category shown in Figure 2d.

26M WT *versus* 3M WT

	No.	Ingenuity Canonical Pathways	*p*-value	Ratio	Molecules
II	1	Calcium Signaling	1.91E-06	3.37E-02	Calr, Tnnc2, Casq1, Trdn, Myl1, Serca1
I	2	Unfolded protein response	5.01E-06	7.41E-02	Calr, Grp94, P4hb, Bip
	3	Endoplasmic Reticulum Stress Pathway	1.15E-05	1.43E-01	Calr, Grp94, Bip
	4	14-3-3-mediated Signaling	1.07E-04	3.42E-02	Ywhah, Tubb4b, Tuba4a, Vim
	5	phagosome maturation	2.09E-03	2.50E-02	Calr, Tubb4b, Tuba4a
	6	Epithelial Adherens Junction Signaling	3.63E-03	2.05E-02	Tubb4b, Tuba4a, Myl1
	7	Aldosterone Signaling in Epithelial Cells	4.07E-03	1.97E-02	Cryab, Grp94, Bip
	8	Germ Cell-Sertoli Cell Junction Signaling	4.68E-03	1.88E-02	Map2k6, Tubb4b, Tuba4a
	9	Acute Phase Response Signaling	5.50E-03	1.78E-02	Map2k6, Serpina1, Serpina3
	10	PPARα/RXRα Activation	6.17E-03	1.69E-02	Map2k6, Grp94, Gpd1
	11	Glycerol-3-phosphate Shuttle	6.31E-03	3.33E-01	Gpd1,
IV	12	NRF2-mediated Oxidative Stress Response	6.46E-03	1.67E-02	Map2k6, Gstm2, Fth1
	13	ILK Signaling	6.92E-03	1.62E-02	Map2k6, Vim, Myl1
	14	Hypoxia Signaling in the Cardiovascular System	8.32E-03	3.08E-02	Grp94, P4hb
	15	PRPP Biosynthesis I	8.51E-03	2.50E-01	Prps1l3,
	16	Remodeling of Epithelial Adherens Junctions	9.12E-03	2.94E-02	Tubb4b, Tuba4a
	39	TCA Cycle II (Eukaryotic)	4.79E-02	4.35E-02	Idh3g,
III	98	Oxidative Phosphorylation	2.06E-01	9.17E-03	Ndufb11,
	120	Mitochondrial Dysfunction	3.04E-01	5.85E-03	Ndufb11,

3M Cisd2 mKO *versus* WT

	No.	Ingenuity Canonical Pathways	*p*-value	Ratio	Molecules
III	1	Oxidative Phosphorylation	3.16E-09	7.34E-02	Ndufv2, Atp5j2, Atp5l, Ndufb6, Cox7a1, Cox4i1, Ndufa8, Ndufb10
	2	Mitochondrial Dysfunction	5.89E-09	5.26E-02	Hsd17b10, Ndufv2, Atp5j2, Atp5l, Ndufb6, Cox7a1, Cox4i1, Ndufa8, Ndufb10
II	3	Calcium Signaling	8.32E-09	5.06E-02	Calr, Myh4, Tnni2, Tnnt3, Tnnc2, Prkar2a, Ryr1, Acta1, Casq2
I	4	Unfolded protein response	1.02E-06	9.26E-02	Calr, Grp94, P4hb, Canx, Bip
	5	Endoplasmic Reticulum Stress Pathway	4.68E-05	1.43E-01	Calr, Grp94, Bip
	6	Lipid Antigen Presentation by CD1	8.91E-05	1.15E-01	Calr, ERp57, Canx
	7	Antigen Presentation Pathway	2.82E-04	7.89E-02	Calr, ERp57, Canx
	8	Isoleucine Degradation I	9.77E-04	1.43E-01	Hsd17b10, Bcat2
	9	Cellular Effects of Sildenafil (Viagra)	9.77E-04	3.08E-02	Myh4, ERp57, Prkar2a, Acta1
	10	Valine Degradation I	1.66E-03	1.11E-01	Bcat2, Aldh6a1
	11	Protein Kinase A Signaling	2.04E-03	1.53E-02	Myh4, Tnni2, ERp57, Prkar2a, Ryr1, Ttn
III	12	TCA Cycle II (Eukaryotic)	2.69E-03	8.70E-02	Idh3a, Idh3b
	13	Glycolysis I	3.16E-03	8.00E-02	Tpi1, Pgam2
	14	β-alanine Degradation I	6.76E-03	5.00E-01	Aldh6a1,
	15	Netrin Signaling	7.59E-03	5.13E-02	Prkar2a, Ryr1
	16	Methylglyoxal Degradation I	1.00E-02	3.33E-01	Glo1,
	18	Glutathione Redox Reactions II	1.35E-02	2.50E-01	ERp57
IV	48	Glutathione Redox Reactions I	7.08E-02	4.55E-02	Gpx3
	72	NRF2-mediated Oxidative Stress Response	1.37E-01	1.04E-02	Gstm2, Acta1
